# Rigosertib ameliorates the effects of oncogenic KRAS signaling in a murine model of myeloproliferative neoplasia

**DOI:** 10.18632/oncotarget.26735

**Published:** 2019-03-08

**Authors:** Stacey J. Baker, Stephen C. Cosenza, M.V. Ramana Reddy, E. Premkumar Reddy

**Affiliations:** ^1^ Department of Oncological Sciences, Icahn School of Medicine at Mount Sinai, New York, NY 10029, USA; ^2^ Department of Pharmacology and Systems Therapeutics, Icahn School of Medicine at Mount Sinai, New York, NY 10029, USA

**Keywords:** rigosertib, RAS, hematopoiesis, myeloproliferative disorder

## Abstract

Aberrant signaling triggered by oncogenic or hyperactive RAS proteins contributes to the malignant phenotypes in a significant percentage of myeloid malignancies. Of these, juvenile myelomonocytic leukemia (JMML), an aggressive childhood cancer, is largely driven by mutations in *RAS* genes and those that encode regulators of these proteins. The *Mx1-cre kras^+/G12D^* mouse model mirrors several key features of this disease and has been used extensively to determine the utility and mechanism of small molecule therapeutics in the context of RAS-driven myeloproliferative disorders. Treatment of disease-bearing KRAS^G12D^ mice with rigosertib (RGS), a small molecule RAS mimetic that is in phase II and III clinical trials for MDS and AML, decreased the severity of leukocytosis and splenomegaly and extended their survival. RGS also increased the frequency of HSCs and rebalanced the ratios of myeloid progenitors. Further analysis of KRAS^G12D^ HSPCs *in vitro* revealed that RGS suppressed hyperproliferation in response to GM-CSF and inhibited the phosphorylation of key RAS effectors. Together, these data suggest that RGS might be of clinical benefit in RAS-driven myeloid disorders.

## INTRODUCTION

Somatic mutations of *RAS* genes are present in approximately 5-40% of hematological malignancies and often arise as secondary events that cooperate with other driver mutations [1,2]. In addition to mutation of *RAS* genes themselves, mutation of genes such as *PTPN11* or *NF1* often result in a loss of negative regulatory cues and ultimately lead to hyperactivation of RAS-driven signaling [1,2]. Of the myeloid malignancies that harbor *RAS* mutations or exhibit abnormally high levels of RAS activity, juvenile myelomonocyitc leukemia (JMML), an aggressive and rare childhood cancer [3], almost invariably (~90%) presents with driver mutations in *KRAS*, *NRAS* or other genes encoding RAS pathway regulatory proteins [4, 1,5]. The high frequency of these genomic alterations suggests that targeting RAS signaling, either by inhibiting RAS proteins themselves, their effectors, or regulators, might be an effective strategy to combat this and other myeloid malignancies that are RAS pathway-dependent.

Rigosertib (RGS) is a small molecule RAS mimetic [6] that is currently in phase II and III clinical trials for high-risk myelodysplastic syndrome (MDS) either as a single agent or in combination with hypomethylating agents (HMAs) [7–9]. Previous studies by us and others have shown that treatment of MDS cell lines and primary bone marrow isolated from MDS patients with RGS resulted in the induction of apoptosis as well as inhibition of RAF1 and AKT phosphorylation at residues that are critical for RAS- and PI3K-driven signaling [10–12]. These pre-clinical data, combined with the agent's safety profile revealed in clinical trials [10, 13], suggest that RGS might be an effective therapeutic in hematological malignancies that exhibit altered RAS-driven signaling and for those where there is not already a perceived clinical benefit [7].

To further examine effects of RGS in RAS-dependent myeloid disorders, we utilized the *Mx1-cre kras^+/LS-LG12D^* mouse model which phenocopies many key aspects of JMML. These mice develop of an aggressive and lethal myeloproliferative neoplasm (MPN) with rapid onset and present with severe anemia, hepatosplenomegaly and leukocytosis [14]. Here, we present data demonstrating that treatment with RGS improves the disease burden in MPN-bearing animals. Our studies show that RGS-treated mice show improvements in complete blood counts and a reduction in the degree of splenomegaly due to a decrease in erythroid cells that accumulate in the spleen. Importantly, we also show that treatment with RGS resulted in a clear survival benefit, suggesting that this compound might be useful in the treatment of myeloid disorders.

## RESULTS

### Effect of rigosertib on KRAS^G12D^-driven myeloproliferative neoplasia

**Figure 1:**
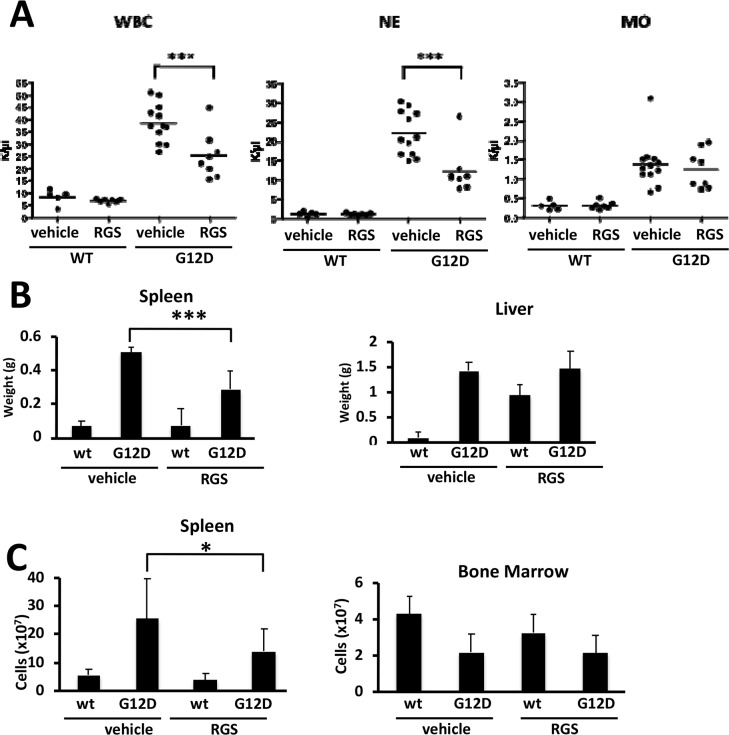
Effects of rigosertib on K-RAS^G12D^-driven myeloproliferative neoplasia **(A)** Complete blood counts of wild-type (WT) and K-RAS^G12D^ (G12D) mice treated with vehicle (PBS) or rigosertib (RGS). WBC: white blood cells; NE: neutrophils; MO: monocytes. **(B)** Weights of spleens and livers isolated from wild-type and K-RAS^G12D^ mice treated with vehicle or RGS. **(C)** Total number of cells isolated from the spleen and bone marrow of wild-type and K-RAS^G12D^ mice treated with vehicle or RGS. All values represent mean ± SD. n=5-12 mice per genotype and group. ^*^p≤0.05; ^***^p≤0.0005.

To determine whether rigosertib (RGS) reduces the disease burden in RAS-dependent myeloproliferative neoplasias (MPNs), *Mx1cre-Kas^+/G12D^* mice [14] were treated with a single dose of polyinosinic:polycytidylic acid (pIpC) to induce KRAS^G12D^ expression in the hematopoietic compartment and the disease allowed to progress over a 14-day period. Complete blood counts performed at this time showed that MPN phenotype was readily evident, as animals presented with marked leukocytosis in the peripheral blood as well as organomegaly of both the liver and spleen (Figure 1A and 1B). *Mx1cre-Kas^+/G12D^* mice treated with RGS [6] over this 2-week period had reduced white blood cell counts (WBCs), with a reduction in neutrophil counts being largely responsible for the overall decrease in WBCs (Figure 1A and data not shown). Monocytosis, which is pronounced in this model [14] and a characteristic feature of JMML and chronic myelomonocytic leukemia (CMML) [2,3,5], persisted in RGS-treated animals.

Further examination of the livers and spleens of vehicle and RGS-treated *Mx1cre-Kas^+/G12D^* mice revealed that while the livers of animals in both treatment groups remained enlarged at the end of the 2-week treatment period, the degree of splenomegaly in RGS-treatment animals was significantly reduced in terms of both organ weight and overall cell number (Figure 1B and 1C, respectively). The cellularity of the bone marrow (BM), which is reduced as a function of KRAS^G12D^ expression [13, 14], was not improved with RGS treatment (Figure 1C).

### Rigosertib suppresses extramedullary erythropoiesis in the spleens of KRAS^G12D^ mice

**Figure 2:**
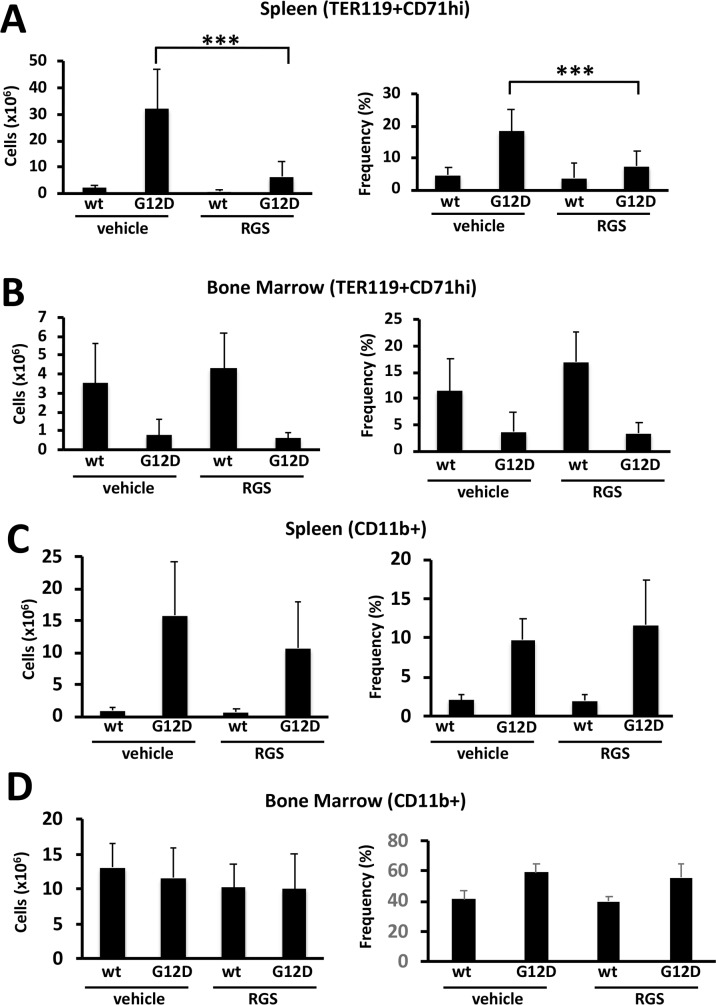
Rigosertib modulates KRAS^G12D^-dependent erythroid and myeloid phenotypes *in vivo* **(A)** Total number (left panel) and frequency (right panel) of TER119^+^CD71^hi^ erythroid progenitors in the spleens and **(B)** bone marrow of wild-type (wt) and K-RAS^G12D^ mice treated with vehicle or RGS. (**C)** Total number (left panel) and frequency (right panel) of CD11b^+^ myeloid cells in the spleens and **(D)** bone marrow of wild-type (wt) and K-RAS^G12D^ mice treated with vehicle or RGS. All values represent mean ± SD. n=5-12 per population for each genotype and group. ^***^p≤0.0005.

Extramedullary hematopoiesis often occurs in myeloproliferative disorders and is recapitulated in the *Mx1cre-Kas^+/G12D^* model [14, 15]. Flow cytometric analysis of the cell types present in the spleen showed that treatment with RGS predominantly reduced the number and frequency of TER119^+^CD71^hi^ cells (Figure 2A); the number of mature myeloid cells was also reduced in the spleens of RGS-treated animals by more than 30% (Figure 2C), although this difference was slightly less than significant (p=0.057) and did not translate into a reduction in the frequency of these cells. Analysis of these erythroid and myeloid populations in the bone marrow also showed that these populations were reduced by 20-25% in RGS versus control-treated animals (Figure 2B and 2D). Together, these results demonstrate that the improvement in splenomegaly induced by short-term RGS treatment is largely due to the selective loss of erythroid progenitors and to a lesser extent, CD11b+ myeloid cells.

### Short-term rigosertib treatment influences the nature of the stem and progenitor compartments in the bone marrow of KRAS^G12D^ mice

**Figure 3:**
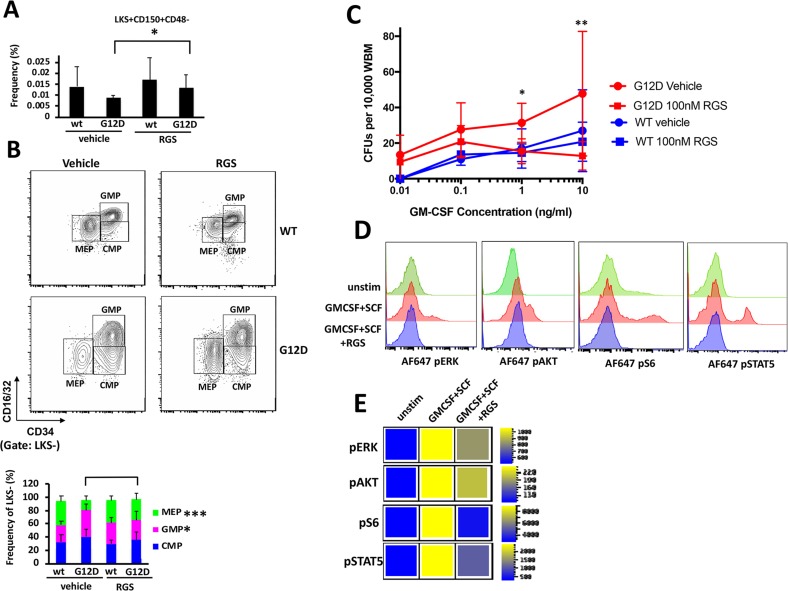
*In vitro* and *in vivo* effects of rigosertib on KRAS^G12D^ hematopoietic stem cells and progenitors **(A)** Frequency of LKS^+^CD150^+^CD48^−^ HSCs and **(B)** LKS- myeloid progenitors in the bone marrow ofwild-type (wt) and K-RAS^G12D^ mice treated with vehicle or RGS. Representative contour plots showing CMP, GMP and MEP populations in K-RAS^G12D^ mice are shown (top). All numerical values represent mean ± SD. n=5-12 per population for each genotype and group. **(C)** Whole bone marrow isolated wild-type and K-RAS^G12D^ mice was plated in duplicate in methylcellulose in the presence of the indicated concentrations of GM-CSF and RGS where indicated. Colonies were enumerated 7 days post-plating. n=3 for each genotype and treatment.^*^p≤0.05. ^**^p≤0.005. **(D)** Phosphorylation status of ERK, AKT, ribosomal S6 and STAT5 in unstimulated (unstim) and SCF/GM-CSF stimulated Lin- primary K-RAS^G12D^ bone marrow treated with vehicle or RGS. Representative histograms and **(E)** heatmaps depicting median fluorescence intensity (MFI) for one representative experiment (out of 3) are shown.

Previous studies have shown that the MPN that develops in *Mx1cre-Kas^+/G12D^* originates in the hematopoietic stem cells (HSCs) within the bone marrow [16–18]. A more detailed examination of the bone marrow revealed that while KRAS^G12D^-induced abnormalities in cellularity were not significantly improved in RGS-treated mice (Figure 1C), the frequency of Lin-Sca1+ckit+ (LKS+) CD150+CD48- HSCs, which are reduced as a consequence of KRAS^G12D^ expression [16, 18, 19], was slightly, but significantly increased in RGS-treated animals (Figure 3A). The abnormal shift to a bias in granulocyte-monocyte lineage progenitors at the expense of those of the erythroid lineage that is conferred by KRAS^G12D^ expression [16, 17] in the RGS-treated group was similar to that observed in wild-type animals, whereby the frequency of megakaryocyte/erythrocyte precursors (MEPs) (Lin-Sca1-cKit+CD34^lo^CD16/32[FcγR]^lo^) and granulocyte/macrophage precursors (GMPs) (Lin-Sca1-cKit+CD34^+^CD16/32[FcγR]^hi^) were increased and reduced, respectively (Figure 3B).

We also assessed whether hypersensitivity of myeloid progenitors to GM-CSF, which is a hallmark of JMML and CMML [2,3], was sensitive to the effects of RGS. For this study, whole bone marrow was isolated from pIpC-treated wild-type and *Mx1cre-Kas^+/G12D^* mice and plated in methylcellulose in the presence of increasing concentrations of granulocyte-macrophage stimulating factor (GM-CSF) and RGS where indicated. Figure 3C shows that as expected, the colony forming units (CFUs) that developed from KRAS^G12D^ bone marrow were hypersensitive to GM-CSF and grew at low concentrations of this cytokine, whereas no CFUs were detectable in cultures derived from wild-type bone marrow. Treatment with RGS suppressed the formation of CFUs in the presence of RGS as a function of GM-CSF concentration in KRAS^G12D^ cultures, and at higher concentrations (>1ng/ml), the number of CFUs was equivalent to those formed by wild-type bone marrow.

### Rigosertib inhibits oncogenic RAS-driven signaling in primary KRAS^G12D^ mouse bone marrow

To confirm that RGS inhibited RAS-driven signaling in hematopoietic stem and progenitor cells (HSPCs), whole bone marrow was isolated from pIpC-treated *Mx1cre-Kas^+/G12D^* mice and grown for 16 hours in the presence or absence of RGS. The cells were then stimulated with stem cell factor (SCF) and GM-CSF and subjected to flow cytometric analysis to analyze the phosphorylation status of ERK, AKT and ribosomal S6 (an AKT effector) in the HSPC-enriched lineage-negative (Lin-) compartment. Figures 3D and E show that treatment with RGS attenuated the phosphorylation of both ERK, AKT and S6, proteins whose functions are regulated by RAS [21,22] and are activated in *Mx1cre-Kas^+/G12D^* mice [16]. We also examined the phosphorylation status of STAT5, which has recently been shown to mediate the phenotypes observed in mutant and hyperactive K- and NRAS-driven hematopoietic phenotypes in animal models [16, 19, 23- 26] and is often aberrantly activated in JMML and other myeloid malignant cells treated with low concentrations of GM-CSF [25]. Treatment with RGS also inhibited cytokine-induced STAT5 phosphorylation in Lin-cells (Figure 3D and E), providing confirmatory evidence that this compound blocks multiple facets of RAS-driven signaling.

### Treatment with rigosertib improves survival in KRAS^G12D^ mice with myeloid neoplasia

**Figure 4:**
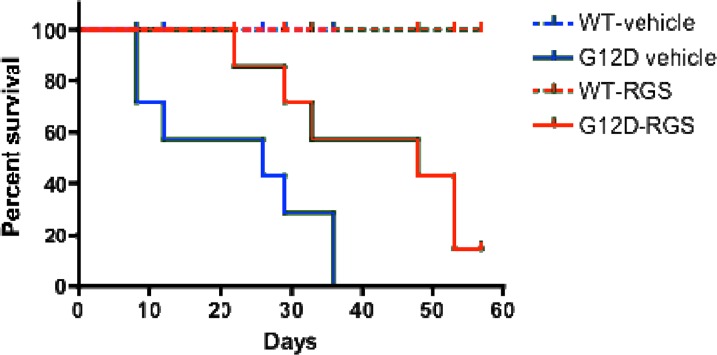
Treatment with rigosertib improves survival in MPN-bearing K-RAS^G12D^ mice Wild-type and *Mx1-Cre KRAS^G12D^* mice were treated once daily with vehicle (PBS) or 100mg/mg rigosertib as described in the methods section. Median survival was 26 days versus 48 days for the vehicle and rigosertib-treated groups, respectively, as estimated by Kaplan-Meier survival analysis. p=0.028 as calculated by the log rank test (n=8 and 7 mice per wild-type and K-RAS^G12D^cohorts, respectively).

To determine if the phenotypic improvements observed in RGS-treated mice might enhance survival, we treated cohorts of wild-type and KRAS^G12D^ mice with vehicle or RGS and monitored these animals over a 2-month period. As seen in Figure 4, while the KRAS^G12D^ vehicle-treated animals succumbed to the effects of MPN at a median of 26 days, RGS-treated mice survived significantly longer, with a median survival of 48 days. Of the 6 RGS-treated animals that eventually succumbed to a lethal myeloproliferative disorder, 5 simultaneously developed T-cell acute lymphoblastic leukemia (T-ALL)/ thymic lymphoma with a predominance of CD4+CD8+ and CD8+ cells (data not shown) [17, 18]. This observation is similar to those seen with MPN-bearing KRAS^G12D^ mice that have been treated with MEK, PI3K or AKT small molecule inhibitors, whereby this non-myeloid malignancy appears to be the primary cause of death in a small percentage of KRAS^G12D^ mice treated with those targeted therapeutics [19, 20]. However, unlike these inhibitors which normalize CBCs and prevent MPN-induced lethality, treatment with RGS appears to delay disease progression as analysis of the hematopoietic compartment in moribund RGS-treated animals displayed the phenotypic characteristics associated with KRAS^G12D^-driven MPN (data not shown).

## DISCUSSION

Here, we present data describing the effects of RGS, a small molecule RAS mimetic [6], as a therapeutic agent in a pre-clinical mouse model of KRAS^G12D^-driven MPN. Previous studies using compound genetically modified mouse models and small molecule inhibitors have highlighted the utility of inhibiting downstream effectors of RAS proteins in the treatment of RAS-driven myeloid malignancies [1, 2, 19, 20, 27–29]. In these instances, overall survival, as well as both disease burden and malignant phenotypes, were dramatically improved, demonstrating that blocking signals that are transmitted downstream of RAS has the potential to be clinically beneficial, even in the absence of elimination of the malignant clone. Our studies show that short-term treatment (2-weeks) with RGS is able to reduce the degree of leukocytosis in myeloproliferative disease (MPD)-bearing KRAS^G12D^ mice as evidenced by reductions in the number of WBCs, particularly neutrophils, in the peripheral blood. This improvement was consistent with a reduction (~30%) in the number of CD11b+ myeloid cells in the spleen and to a lesser extent in the bone marrow (~20%). Although the frequency of these cells remained similar to that of vehicle treated animals, the substantial loss of TER119+ cells in the spleen (discussed below) likely results in a commensurate increase in the frequencies of other populations.

Analysis of the spleen revealed that the severity of splenomegaly was improved by RGS treatment and was largely due to a reduction in the number of TER119^+^CD71^hi^ erythroid progenitors. Although the nature of this response would be considered palliative at best in the absence of improvements in erythropoiesis and anemia in the remainder of the hematopoietic compartment, patients with hematological disorders that are phenocopied here could still achieve clinical benefit from RGS. Ruxolitinib, a JAK1/JAK2 inhibitor which is used for the treatment of myelofibrosis, received approval from multiple agencies due to its ability to reduce symptoms of the disease, including splenomegaly by 35% [30, 31]. Given that RGS-treated KRAS^G12D^ mice survived significantly longer than those treated with vehicle, it is tempting to attribute this to a reduction in spleen volume, possibly in conjunction with improvements in overall peripheral blood count. However, as mentioned in the results section, this response is not durable and the majority of animals treated long-term (2 months) ultimately succumbed to the effects of MPN as well as T-cell leukemia. It should be noted, however, that the dosing regimen used herein is a caveat of the long-term study. We have previously shown that the number and grade of pancreatic intraepithelial neoplastic lesions in *Pdx cre-Kras^+/G12D^* mice were significantly reduced in animals treated twice-daily with 200mg/kg RGS and that the decrease in tumor grade and burden correlated with inhibition of RAS-driven signaling and the induction of apoptosis [6]. Although we did observe phenotypic improvements in our short-term study that utilized the same dose of RGS, mice in the long-term study (Figure 4) were treated once-daily with 100mg/kg RGS to minimize the effects of repeated intraperitoneal injections over a 2-month period. Hence, we were unable to determine the utility of RGS in prolonging survival of this model to the fullest extent.

We also examined the effects of RGS in HPSCs since the MPN in KRAS^G12D^ mice originates in the HSCs and is also manifested in hematopoietic progenitors [16–18]. KRAS^G12D^ expression in the bone marrow results a loss of HSCs, with those that remain having enhanced repopulating ability due to increased cell cycle entry. Cell cycle progression in myeloid progenitors is also enhanced, although these cells are unable to initiate leukemias in competitive bone marrow transplantation assays [17, 18]. The frequency of HSCs was significantly increased in RGS-treated animals compared to the vehicle-treated cohort, suggesting that RGS might have the ability to alter the behavior of the stem cell pool. It is, however, unclear whether the neoplastic behavior of these cells is altered in the absence of data from bone marrow transplantation studies. The percentage of MEPs, which is decreased as a function of KRAS^G12D^ expression [16, 17], was also restored to nearly normal levels in response to RGS treatment and was associated with a concomitant decrease in the abnormally high frequency of GMPs in these animals. Interestingly, hyperactivation of STAT5 in *Mx1cre-Nas^+/G12D^* mice is mainly due to expansion of granulocytic-monocytic progenitors to GM-CSF [26] as well as proliferating and self-renewing HSCs [23]. The mechanism by which mutant RAS isoforms activate STAT5 is not understood, and it is unclear if these mechanisms also apply to KRAS^G12D^-driven MPDs. Given that the perceived phenotypic corrections in HSPCs within the bone marrow do not always translate into improvements in all hematopoietic tissues, use of RGS in combination with other agents that might synergize with and enhance its effect in these cell types might be of clinical benefit.

## MATERIALS AND METHODS

### Mice

*Kras+/LSLG12D* (stock 008179) and *Mx1-Cre* (stock 003556) mice were purchased from The Jackson Laboratory. Breeding and experiments were performed under protocols approved by the Icahn School of Medicine at Mount Sinai's Institutional Animal Care and Use Committee according to federal and institutional guidelines and regulations

Polyinosinic:polycytidylic acid (pIpC) (Sigma) was resuspended at a concentration of 2.5mg ml^-1^ in sterile Dulbecco's PBS (D-PBS). Mice were injected intraperitoneally (ip) with a single dose of pIpC (250μg) at 4-6 weeks of age. Treatment with vehicle (sterile PBS) or GMP-grade rigosertib (RGS) (Onconova Therapeutics, Inc.) was initiated 10-14 days post-pIpC treatment. RGS was administered twice daily via ip injection 5 days per week a dose of 200mg/kg for short-term (2 weeks) studies as previously described by us [6]. Mice treated long-term (2 months) were treated once daily with a dose of 100mg/kg of RGS on a 5 day on, 2 days off schedule. RGS was freshly dissolved in PBS at the time of each injection for all studies. Tissues were harvested at the times indicated. Complete blood counts were measured using a Hemavet 950 multi-species hematology system (Erba Diagnostics).

### Colony formation assays

1×10^4^ whole bone marrow cells isolated from pIpC-treated *Kras^+/G12D^* or wild-type mice were plated in 1.5ml of Methocult M3231 (Stemcell Technologies) supplemented with the indicated concentrations of GM-CSF (Peprotech) and RGS in a non-treated 35mm dish in duplicate. Cells were cultured for 7 days before colonies were scored.

### Flow cytometry

Single cell suspensions were prepared in phenol red-free RPMI supplemented with 2% heat-inactivated fetal bovine serum (FBS). Defined numbers of cells were stained on ice for 30 min. (or 90 min for CD34), washed and then subjected to flow cytometric analysis as described by us [32]. Fluorochrome-conjugated antibodies used for staining are as follows: lineage (Lin) cocktail [Gr1 (RB6-8C5), CD11b (M1/70), CD3 (17A2), B220 (RA3-6B2), TER119 (TER-119)] conjugated to either FITC or e450; c-kit/CD117 (2B8) conjugated to APC-H7 or APC-Cy7; Sca-1 (D7) conjugated to either PE-Cy7 or PerCP-Cy5.5; PECy7 CD48 (BCMI); CD34 (RAM34) conjugated to either FITC or e450; CD16/32 (93) conjugated to PerCP-Cy5.5, Alexa Fluor 647 or APC, CD150 PerCPCy5.5 or APC (TC15-12F12.2); CD71 (R17 217.1.4) conjugated to PE, TER119 (TER-119) conjugated to either e450 or PerCP-Cy5.5, Gr1 (RB6-8C5) conjugated to PE and CD11b (M1/70) conjugated to PE-Cy7. Antibodies were purchased from Thermo Scientific, BioLegend or BD Biosciences.

Intracellular staining and subsequent flow cytometric analysis of phosphorylated proteins in RGS-treated primary bone marrow was performed as follows: single cell suspensions were prepared in Iscove's Modified Dulbecco's medium supplemented with 10% heat-inactivated FBS, seeded at a density of 1×10^7^ cells/ml in the presence of vehicle (PBS) or 2μM RGS and grown for 16 hours at 37°C under humidified conditions and 5% CO_2_. The cells were then stained with Lin cocktail antibodies conjugated to PE for 15 minutes at 37°C prior to stimulation with 100ng/ml SCF and 10ng/ml GM-CSF (Peprotech) for 5 min where indicated. Fixation was performed using 1X Lyse/Fix buffer (BD Biosciences) according to the manufacturer's instructions. Cells were then permeabilized on ice for 30 min using Perm Buffer III (BD Biosciences) and washed extensively using PBS prior to staining with the indicated phospho-antibodies on ice for 1 hr. The samples were then washed with PBS and subjected to flow cytometric analysis on the day of staining. AF647-conjugated phospho-specific antibodies directed against phospho-p44/42 MAPK Thr202/Tyr204 (E10), phospho-STAT5 Tyr694 (C71E5), pAKT Ser473 (D9E) and phospho-S6 ribosomal protein (Ser235/236) (D57.2.2E) were purchased from Cell Signaling Technology.

Data were acquired using an LSRFortessa X-20 or FACS Canto (BD Biosciences) and analyzed using FlowJo v10 software (Treestar). Bone marrow from 2-3 *Kras^+/G12D^* mice was pooled prior culturing for each independent experiment.

### Statistical analysis

All data were analyzed using Prism 7 (GraphPad). Kaplan-Meier survival estimates were analyzed using the log-rank test. Statistical analysis of differences in CBC numbers as well as population subsets in the bone marrow, spleen, liver and peripheral blood were performed using a standard, unpaired, two-tailed Student's *t* test. Data are graphed as mean ± SD. Results are considered significant at p≤0.05.

## Abbreviations

BM: bone marrow
RGS: rigosertib
HMA: hypomethylating agent
pIpC: polyinosinic:polycytidylic acid
MPN: myloproliferative neoplasm
MPD: myeloproliferative disorder
HSPC: hematopoietic stem and progenitor cell
HSC: hematopoietic stem cell
Lin: lineage
LKS: Lin, c-kit, Sca-1
CFU: colony forming unit
GM-CSF: granulocyte-macrophage stimulating factor
JMML: juvenile myelomonocyitc leukemia
CMML: chronic myelomonocytic leukemia
